# Forecast of electric vehicle uptake across counties in England: Dataset from S-curve analysis

**DOI:** 10.1016/j.dib.2021.107662

**Published:** 2021-12-01

**Authors:** Katherine A. Collett, Sivapriya M. Bhagavathy, Malcolm D. McCulloch

**Affiliations:** Energy and Power Group, Department of Engineering Science, University of Oxford, Parks Road, Oxford OX1 3PJ, United Kingdom

**Keywords:** Electric vehicles, Forecast, Uptake, S-curve analysis, EVs

## Abstract

Regional data from the UK Government's Department for Transport has been analyzed to produce a forecasted dataset of the uptake of electric vehicles (EVs) within Counties of England to the first quarter of the year 2100 using an S-curve methodology. This data includes all vehicles, not just cars. The historic proportion of electric vehicles in the fleets of these regions is calculated using data from 2011 Q4 to 2021 Q1. This data is then analyzed using SCATE, the **S**-**C**urve **A**doption **T**ool for **E**Vs to forecast the future proportion of electric vehicles in these Counties. Two data tables are presented: the reformatted historic data and the data from the S-curve analysis. Data is also presented for the collective UK.

## Specifications Table

**Table:** 

Subject	Renewable Energy, Sustainability and the Environment
Specific subject area	Forecast of the uptake of electric vehicles within the U.K. using a S-curve methodology
Type of data	Table
How data were acquired	Data was acquired by analysing primary data published by the UK Government's Department for Transport in order to forecast future uptake of electric vehicles using SCATE.
Data format	Analyzed
Parameters for data collection	Primary data was from the UK Government's Department for Transport for the UK from 2011 Q4 to 2021 Q1. The datasets include: • The number of battery electric vehicles licensed at the end of the quarter by local authority; • The number of licensed vehicles at the end of the quarter by postcode district and body type. S-curve forecasts data is presented for each English County and the collective UK until the year 2100.
Description of data collection	In order to structure the data into Counties, look-up tables were needed to convert the data from Local Authorities and postcode districts to county. These were created from the below data sources. S-curve analysis [1] was conducted using the historical proportion of electric vehicles in a County between 2011 Q4 and 2021 Q1.
Data source location	Data presented for Counties in England and the collective UK. Primary data came from: UK Government's Department for Transport provided data on the number of battery electric vehicles (Table VEH0132b) and the number of licenced vehicles (Table VEH0122). Available at: https://www.gov.uk/government/collections/transport-statistics-great-britain[3] UK Government's Office for National statistics provided data from which the look-up table for local authorities within England to Counties was created. Available at: https://geoportal.statistics.gov.uk[4] The look-up table for postcode districts within each English County was developed from data available for download at: https://www.doogal.co.uk/counties.php[5]
Data accessibility	On GitHub at: https://github.com/EPGOxford/SCATE and via Zenodo: https://zenodo.org/badge/latestdoi/432239559[2]. The data is saved in the following two files: Percent_cumulative_EVs_historic_2021_Q1.xlsx Percent_cumulative_EVs_S_curve_2021_Q1.xlsx Instructions for accessing these data: Please create a GitHub account and visit the link above to download the data. The code to run SCATE can also be accessed at this link.

## Value of the Data

• Simulating S-curves to forecast EV uptake, transforms the historic primary data into useful future-looking data for planning.
• Reviewing the simulated S-curve data will be important in preparing for the uptake of electric vehicles across Counties in England.
• Policy makers, local authorities, businesses, and academics can all benefit from using these S-curve data in their analyzes and planning for the increasing number of electric vehicles.
• Further insights may be gathered from using the S-curve data to analyze the regional future demand for electric vehicle charge points, estimating future electric vehicle energy consumption in a County, and for infrastructure planning, among other applications.

## 1 Data Description

The data files:

**Percent_cumulative_EVs_historic_2021_Q1.xlsx** is the data file showing the historic data reformatted from the UK Government's Department for Transport. This includes the total number of vehicles, the number of EVs, and the calculated historic percent of vehicles which are battery electric for each quarter (between 2011 Q4 and 2021 Q1) in each County.

**Percent_cumulative_EVs_S_curve_2021_Q1.xlsx** is the simulated S-curve data, i.e. the original data presented. This shows the forecast S-curve data based on the historic data for each County in England up to 2100 Q1.

Files relevant for running the analysis tool SCATE:

**main.py** is the primary code file to be run in python. This is where the files containing the primary data and look-up tables described below are defined and the analysis period for SCATE is set. This file then calls on other sub-modules to re-structure the primary data and to perform the S-curve analysis.

**dataextract.py** is a sub-module to main.py. This module extracts data from the primary data sources and uses the look-up tables to group the historic data by County.

**analyzer.py** is a sub-module to main.py. When this module Is called, the S-curve analysis is conducted, to assess future adoption of EVs in each County.

**analyservaribales.py** is a sub-module to main.py. This file defines variables necessary for analyzer.py to run.

**LA2County.xlsx** is the look-up table used to convert the primary data that is grouped by Local Authority to instead be grouped by County, derived from Office for National Statistics data [4].

**CountyDistricts.xlsx** is the look-up table used to convert the primary data that is grouped by Postcode District to instead be grouped by County, derived from data available on doogal [5].

To run SCATE [2], the user will need files containing the primary data, in this case **veh0132.xlsx** (May 2021) and **veh0122.xlsx** (May 2021) which are included and can also be downloaded from the UK Government's Department for Transport website [3].

## 2 Experimental Design, Materials and Methods

The primary data files from the UK Government's Department for Transport were processed using the python analysis tool named SCATE, developed by the authors. SCATE is the **S**-**C**urve **A**doption **T**ool for **E**Vs and is available on GitHub at: https://github.com/EPGOxford/SCATE.

The primary data files were veh0122.xlsx (May 2021), the total number of licensed vehicles at the end of the quarter, and veh0132.xlsx (May 2021), the total number of battery EVs licenced at the end of each quarter. The data in the files is aggregated in two different ways: by Postcode District and Office of National Statistics Local Authority Code, respectively. In order to regroup the data into County categories, the look-up tables CountyDistricts.xlsx and LA2County.xlsx were used as keys to group Postcode Districts and Local Authorities by County. The number of EVs in each County is then divided by the total number of vehicles in that same County to give the percentage of EVs. Where a postcode spans two Counties, the total number of registered vehicles within that postcode are assigned to both counties. This ensures that when data for each County is assessed, the proportion of EVs will be an underestimate. However, it means that this data cannot be summed to represent England and users should be aware. Thus, the historic proportion of EVs in a County were calculated.

Once the historic proportion of EVs in a County had been calculated, this data was used as an input for S-curve analysis, conducted in python. The following equation was used:

$$y\left(t\right)=\;\frac{1}{1+\alpha e^{-\beta t}},$$

where y is the proportion of EVs in the fleet, t is the time and α,β are parameters that determine the speed and shape of transition. The limits of the equation are 0 and 1.

S-curve analysis was considered appropriate as it is the uptake pattern often seen for emerging technologies, as was the case for the initial adoption of the internal combustion engine vehicle.

## Ethics Statement

The acquisition of this data conformed to Elsevier's ethics in publishing.

## CRediT authorship contribution statement

**Katherine A. Collett:** Methodology, Software, Investigation, Validation, Writing – original draft, Writing – review & editing. **Sivapriya M. Bhagavathy:** Conceptualization, Methodology, Software, Funding acquisition. **Malcolm D. McCulloch:** Conceptualization, Supervision, Funding acquisition.

## Declaration of Competing Interest

The authors declare that they have no known competing financial interests or personal relationships which have or could be perceived to have influenced the work reported in this article.
